# Retinal texture biomarkers may help to discriminate between Alzheimer’s, Parkinson’s, and healthy controls

**DOI:** 10.1371/journal.pone.0218826

**Published:** 2019-06-21

**Authors:** Ana Nunes, Gilberto Silva, Cristina Duque, Cristina Januário, Isabel Santana, António Francisco Ambrósio, Miguel Castelo-Branco, Rui Bernardes

**Affiliations:** 1 Coimbra Institute for Biomedical Imaging and Translational Research (CIBIT), Health Sciences Campus, Polo III, Azinhaga de Santa Comba, Coimbra, Portugal; 2 Institute of Nuclear Sciences Applied to Health (ICNAS), Health Sciences Campus, Polo III, Azinhaga de Santa Comba, Coimbra, Portugal; 3 Faculty of Medicine, University of Coimbra, Health Sciences Campus, Polo III, Azinhaga de Santa Comba, Coimbra, Portugal; 4 Movement Disorders Clinic, Department of Neurology, Centro Hospitalar e Universitário de Coimbra (CHUC), Praceta Prof. Mota Pinto, Coimbra, Portugal; 5 CNC.IBILI Consortium, Health Sciences Campus, Polo III, Azinhaga de Santa Comba, Coimbra, Portugal; 6 Dementia Clinic, Department of Neurology, Centro Hospitalar e Universitário de Coimbra (CHUC), Praceta Prof. Mota Pinto, Coimbra, Portugal; 7 Center for Neuroscience and Cell Biology (CNC), Zoology Department, University of Coimbra, Coimbra, Portugal; 8 Coimbra Institute for Clinical and Biomedical Research (iCBR), Health Sciences Campus, Polo III, Azinhaga de Santa Comba, Coimbra, Portugal; Massachusetts Eye & Ear Infirmary, Harvard Medical School, UNITED STATES

## Abstract

A top priority in biomarker development for Alzheimer’s disease (AD) and Parkinson’s disease (PD) is the focus on early diagnosis, where the use of the retina is a promising avenue of research. We computed fundus images from optical coherence tomography (OCT) data and analysed the structural arrangement of the retinal tissue using texture metrics. We built clinical class classification models to distinguish between healthy controls (HC), AD, and PD, using machine learning (support vector machines). Median sensitivity is 88.7%, 79.5% and 77.8%, for HC, AD, and PD eyes, respectively. When the same subject has the same classification for both eyes, 94.4% (median) of the classifications are correct. A significant amount of information discriminating between multiple neurodegenerative states is conveyed by OCT imaging of the human retina, even when differences in thickness are not yet present. This technique may allow for simultaneously diagnosing Alzheimer’s and Parkinson’s diseases.

## Introduction

Diagnosis of both Alzheimer’s disease (AD) and Parkinson’s disease (PD) remains a major challenge. Simple diagnostic tests serving as biomarkers for tracking the onset and progression of both diseases would be extremely valuable.

AD affects 5 to 7% of people over the age of sixty [1], and a total of 5.4 million patients in the United States [2]. For PD, the incidence has been reported to be 4.5–19 per 100 000 population, per year [3]; while its age-adjusted prevalence is estimated to range between 72 and 259 per 100 000. In the US, the direct costs of health and social care associated with dementia patients have been estimated to reach USD 100 billion per year [3]. Similarly, for PD patients the total annual cost is more than the double of that of the control population [3].

Even though epidemiological evidence suggests a potential reduction in dementia incidence in recent years, its prevalence, according to Shah and co-workers [4], is expected to increase in the coming decades due to population ageing. The impact of dementia in patients and the overall society was acknowledged in the First WHO Ministerial Conference on Global Action Against Dementia (March 2015), bringing together, among others, representatives from 80 WHO Member States and four UN agencies [4]. On the top priorities in the domain of diagnosis, one can find the research aiming to promote timely and accurate diagnosis of dementia through the development and validation of simple riskless biomarkers that could even be used in prodromal disease stages. Moreover, the focus on the need to “… *identify similarities and differences between diseases and dementia subtypes, and assess progression from premanifest (presymptomatic) to late-stage diseases*” [4] is stressed in the report. Furthermore, priorities point to the need to diagnose dementia in the primary care practices, thus putting aside the focus on the diagnosis based on brain imaging requiring instrumentation available only at advanced imaging centres, where the access is only possible to a fraction of the population—not to mention the associated costs.

The morphological effects of the neurodegenerative process seem to be similar in the retina and the brain [5]. Therefore, the use of the retina as a window into the brain [6–8] might provide a simple solution for the establishment of reliable image-based biomarkers for neurodegeneration. This concept has been extensively exploited, mostly regarding the onset and progression of AD [9, 10] and PD [11, 12]. Notably, there is published evidence that the retinal structure might be altered in the course of these two neurodegenerative disorders [13–16].

When compared to brain imaging, the easy access to and lower operational cost of the ophthalmological imaging techniques are definite advantages, bringing the diagnosis closer to the primary care practices. Even though optical coherence tomography (OCT) has been extensively used in assessing neurodegenerative disorders, the hallmark has been the use of thickness measurements, either at the individual retinal layer level or in an aggregated fashion. While the thinning of specific layers has been the standard finding for both disorders, some contradictory findings can be found in the literature [9, 17–21]. Particularly in the case of AD, it has recently been reported that the thickness of some of the inner retinal layers undergoes dynamic changes as the disease progresses [22]. Moreover, with the retinal nerve fibre layer (RNFL) and retinal ganglion cell axons being the top candidates for the occurrence of neurodegeneration, it should be kept in mind that the thinning of this layer may occur for other types of dementia beyond AD or PD, such as Lewy body dementia [8].

Recent research [23–28] has pointed towards texture analysis as a promising tool in the study of biomarkers for neurodegenerative diseases. The term “texture analysis” encompasses a wide range of methods that allow for the characterisation of the underlying image patterns [29–31]. Such methods, when applied to images of the human retina, can help identify changes in the structural arrangement of the retina or that of specific retinal layers, both in health and disease.

In the present study, we address these issues by imaging the ocular fundus by OCT of healthy controls (HC), as well as that of patients diagnosed with AD and PD. Using a single age-matched control group and two groups of patients, we were able to develop a classification system based on texture characteristics of fundus images computed from OCT data. This system achieved a significant triple clinical classification accuracy.

## Materials and methods

### Participants

OCT data from 20 patients diagnosed with AD, 28 patients diagnosed with PD and 27 age-matched HC were gathered from the authors’ institutional OCT database. The two studies (AD and PD) from which data were collected were approved by the Ethics Committee of the Faculty of Medicine of the University of Coimbra and were conducted according to the principles stated in the Declaration of Helsinki [32]. Written informed consents were obtained from all participants. Before the inclusion in this study, the AD patients underwent a thorough neuropsychological evaluation process, administered by experienced neurologists. As all these patients scored a value of 1 in the clinical dementia rating (CDR) scale, they were deemed capable of signing the written informed consent themselves.

The AD and PD groups were recruited respectively at the Dementia and the Movement Disorders Units of the Neurological Department of the Centro Hospitalar e Universitário de Coimbra, where they were assessed by experienced neurologists. All participants had been previously imaged using the same OCT device and acquisition protocol on both eyes. Due to the low quality of the signal, four eyes were rejected: two from the AD group and two from the PD group.

For the diagnosis of AD, the standard criteria [33] was used, and patients were extensively characterised using formal neuropsychological evaluation as well as specific cognitive and functional staging scales, including the Montreal cognitive assessment (MoCA) [34, 35]—please refer to S1 Table for the MoCA scores. Patients were further investigated with obligatory laboratory, imaging studies and cerebrospinal fluid (CSF) analysis to exclude other forms of reversible dementia or systemic diseases. According to the main aims of the study, patients were strictly selected with a probable diagnosis of AD supported by positive CSF biomarkers (amyloid-*β*42, total tau and phosphorylated tau) and positive Pittsburgh Compound-B (PiB) and positron emission tomography (PET).

Further details on the methods used for the collection and analysis of CSF and PiB-PET data can be found in [36].

All patients had recently converted to AD from a prior stage of mild cognitive impairment. They were all in mild stage (CDR score = 1), with diagnosis duration ranging from zero to two years (see S1 Table for more detail) and were in a stable condition. Retinal injuries and retinopathies, optic neuropathies secondary to other factors (e.g. glaucoma, diabetes, age-related macular degeneration) and severe visual impairment were exclusion criteria.

The PD patients were assessed using the MoCA, the unified Parkinson’s disease rating scale (UPDRS), and Hoehn and Yahr staging (test scores are available in S2 Table). These patients were diagnosed by a movement disorder neurologist, according to the criteria defined by the UK Parkinsons’s Disease Society Brain Bank [37]. Only patients with ages ranging between 40 and 85 years old were included in the study. Subjects showing signs of advanced dementia, severe depression and history of substance abuse were excluded. Furthermore, a neuro-ophthalmologist assessed the patients and those with intrinsic optic nerve or macula pathology were excluded.

All subjects in the present study were selected towards the best age-match between groups and a balanced distribution by gender within each group. At the time of OCT scan, the PD patients had a diagnosis duration that ranged from one to 19 years (Q1/median/Q3: 2.75/5.00/10.00)—please refer to S2 Table for more detail. These patients’ disease duration is modestly correlated (R^2^ = 0.035) to their age (S1 Fig). Demographic data for all subjects in the current study are presented in Table 1.

**Table 1:** Demographic data of the control and patient groups.

	Healthy Controls	Alzheimer’s Disease	Parkinson’s Disease
N	27	20	28
Age—mean(std) (years)	64.1(7.1)	66.3(6.8)	63.4(6.6)
Age—min(max) (years)	53(75)	54(76)	53(77)
Male(Female)	13(14)	10(10)	13(15)
Right(Left) Eyes	26(27)	20(19)	27(27)
Total Acquisitions	53	39	54

### Imaging protocol

Retinal imaging was performed using the Cirrus SD-OCT 5000 (Carl Zeiss, Meditec, Dublin, CA, USA) and the 512x128 Macular Cube protocol, centred on the macula.

### Data pre-processing

The gathered volumetric data were further processed using the OCT Explorer software (Retinal Image Analysis Lab, Iowa Institute for Biomedical Imaging, Iowa City, IA, USA) to segment eleven retinal layers: the RNFL, ganglion cell layer (GCL), inner plexiform layer (IPL), inner nuclear layer (INL), outer plexiform layer (OPL), outer nuclear layer (ONL), inner segment/outer segment junction, outer segment, outer photoreceptor, subretinal virtual space, and retinal pigment epithelium (RPE). These segmentations were visually examined and manually corrected where necessary.

**Fig 1:**
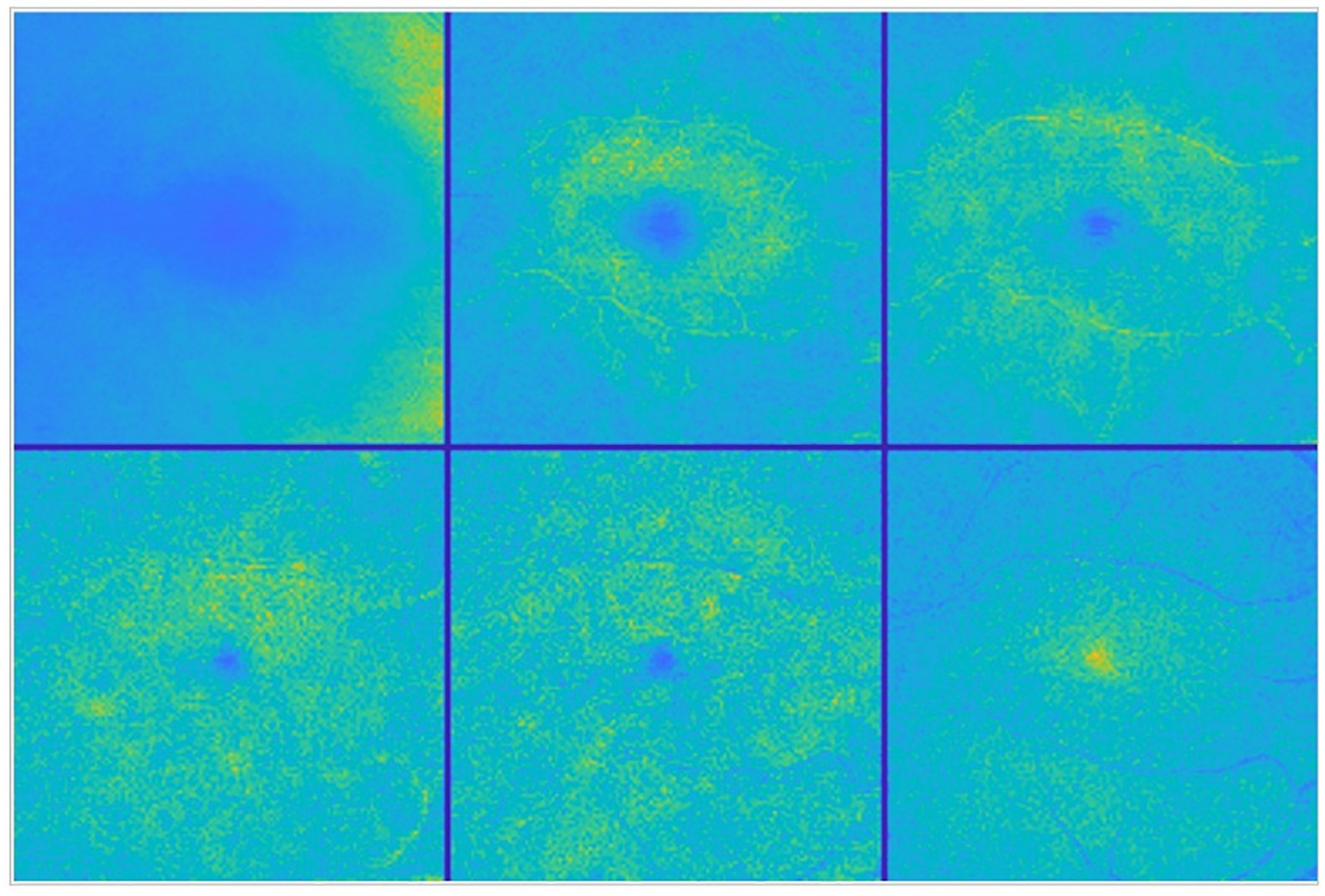
Mean value fundus images. Colour-coded MVF images from the right eye of a healthy control. From left to right and top to bottom: RNFL, GCL, IPL, INL, OPL and ONL layer fundus images.

From each of the six inner retinal layers, the RNFL, the GCL, the IPL, the INL, the OPL, and the ONL, a mean value fundus (MVF) image [38] was computed as the average of the A-scan values between the two retinal layer interfaces defining the layer at study. These six images per eye constitute the data to be further processed and analysed throughout this work. All left eyes were horizontally flipped to match the right ones and to allow metrics to keep the same relative position. Fig 1 shows an example for reference purposes only, where layers were intensity corrected and pseudo-colour coded for ease of visualisation.

### Retinal thickness

The macular thickness was computed for each of the six retinal layers at study in this work, along with the full retinal thickness, i.e. the thickness from the inner limiting membrane to the top of the RPE.

### Texture analysis

There are several methods for retrieving textural features from an image. Frequently, statistical methods such as the gray level co-occurrence matrix (GLCM) [39, 40] are employed. These methods provide insight into the patterns and relative distribution of the image’s intensity values. In the particular case of the GLCM, localised grey-level variations are recognised throughout different directions, and scales, examining pixel pairs iteratively. Alternatively, spectral texture features can be computed. By evaluating spatial frequencies at multiple scales, the wavelet transform has a natural application in texture analysis, where it can help to identify general/coarse texture features that statistical approaches like the GLCM fail to capture [29]. For this reason, wavelet-based texture descriptors have been computed and used in the classification of different types of medical images [41–45]. Both GLCM and wavelet-based texture features were used in this work to capture, respectively, local and global texture information.

**Fig 2:**
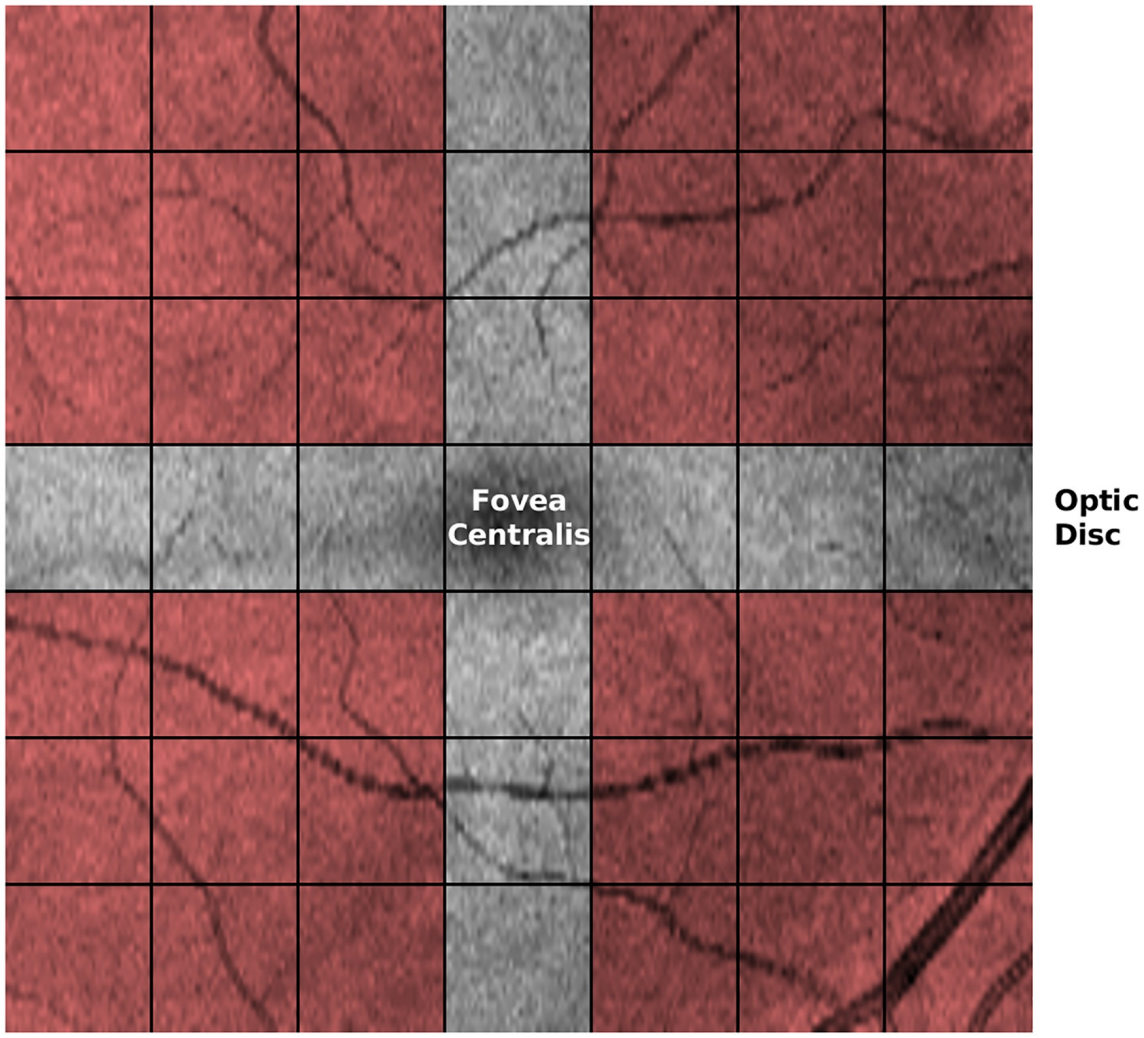
Mean value fundus images’ blocks. Computed fundus image from the volumetric macular cube scan of the right eye of a healthy control subject. Each of the 7x7 blocks show the individually analysed areas which results were later aggregated into larger regions (shaded areas). Image axes are: x-axis (horizontal)—temporal (left) to nasal (right) and y-axis (vertical) superior (top) to inferior (bottom).

Regarding the GLCM, each image was first down-sampled to 128x128 pixels to obtain isotropic sampling in the horizontal and vertical directions and converted to 16 grey-level to limit the size of the GLCM matrix. These images were then split into 7x7 blocks to be independently analysed. The supremum over the different directions of each of the 20 metrics computed from the GLCM, for each block, was chosen as the respective value for the block. This information was later aggregated per quadrant, the superior-temporal, the superior-nasal, the inferior-temporal and the inferior-nasal quadrants. Each quadrant metric is the average of that of the respective 3x3 blocks, leaving out the row and column passing through the fovea (Fig 2). As such, a total of 80 GLCM-based features characterise each layer of the retina.

Concerning spectral texture features, the dual-tree complex wavelet transform (DTCWT) [46] was applied to each of the fundus images. The variance and entropy of the magnitude of the DTCWT complex coefficients were computed for all image subbands, following the approach used previously [47, 48]. Exploratory analysis revealed that the variance alone, when computed for the six directionally selective subbands (±15°, ±45° and ±75°) at decomposition level one, leads to the best performance. These six directional variance values were therefore combined with the 80 GLCM-based features to form the final feature vector, composed of a total of 86 features per layer.

### Classification

The selected method for data classification was the support vector machine (SVM). SVM-based classification [49, 50] is a two-step procedure where, in the first step, SVMs learn the differences between two groups to establish a classification model (training phase). In the second step, this model is used to classify cases not used during the first step. The validity of the model can be assessed (test phase) by using known cases in the second-step and comparing the predicted classifications to the real ones.

Prior to the classification, all features were independently z-score transformed to eliminate differences in scale that may interfere with the system’s performance.

In total, 18 independent binary SVM models (with the radial basis function kernel) were developed, one per each of the six retinal layers at study and between any two of the three possible groups at study: the HC, AD, and PD groups.

Six features were considered for each of the 18 models to achieve the best classification performance while avoiding overtraining, considering the number of cases (eyes) in each of the groups (classes). The identification of the particular set of features for each of the models was performed by resorting to a recursive forward selection procedure. In this approach, a single feature is tested at a time, and the one allowing for the best performance is selected. After that, at a time, each one of the remaining features is considered, and the performance based on the two features (the feature currently being considered, plus the previously selected “best” one) is assessed. The set presenting the best performance is kept, and the process repeats until the best set with the predefined number of features is found.

The performance of each of the classification processes was assessed based on the *k*-fold cross-validation. In this procedure, the dataset at hand is split into *k* sets. The system is then trained with *k*-1 sets and tested on the set left out from the training. The testing consists of predicting the classes where the cases left out from the training would belong to, based on the model developed during the training phase, and comparing the predictions with the known (real) classification. This procedure is repeated *k* times.

For the classification of an eye in one of the three possible classes, a one-versus-one approach was used, along with a voting scheme. As such, for each of the six retinal layers at study, each eye was tested against the model between the control and AD classes, between the control and PD classes and between the AD and PD classes. For each of these three tests, the eye received a vote in one of the two classes under evaluation.

At the end of this procedure, the eye was classified into the most voted class. If all three classes had received the same number of votes, a tie was considered, and the eye was classified as “Unknown”. If two classes received the same number of votes, the final decision was made by considering the result of the classification between those two classes. A diagram illustrating the classification process is shown in S2 Fig.

### Statistical analysis

Based on the classifications of all eyes, using the classification process described above, a 3x4 confusion matrix was built, where the three rows represent the real (known) classes, and the four columns represent the classes according to the data and classification models. The extra “Unknown” class is composed of the cases with ties on the classification.

From the confusion matrix, the sensitivity and specificity were computed for each class: the control, the AD, and PD classes. The system accuracy was also computed.

Additionally, the percentage of participants with both eyes receiving the same classification was determined, along with the percentage of these that were correctly classified.

The last computed parameter was the percentage of eyes in the “Unknown” class, that is, the percentage of eyes where, from the texture point of view, no substantial differences were found between the HC, AD, and PD study populations.

All results were computed using Matlab R2017b (The MathWorks Inc., Natick, MA, USA) running on a personal computer.

## Results

Table 2 presents the results of the full retinal thickness measurement distributions for the three groups. Thickness values are the average thickness within nine regions centred on the fovea. The central region is the circular area of 1000 micrometres in diameter, inner macular areas are within the radii 1000 and 1500 micrometres, and outer macular areas are within the radii 1500 and 3000 micrometres.

**Table 2:** Full-retinal thickness. Sectors mimic the ETDRS thickness map. Inner macular areas are within the radii 500 and 1500 micrometres, and outer macular areas are within the radii 1500 and 3000 micrometres.

	Healthy Controls	Alzheimer’s Disease	Parkinson’s Disease	*p*-value (ANOVA)
Central Subfield	258.2 ± 19.6	254.9 ± 17.9	255.3 ± 23.3	0.6806
Nasal Inner	326.5 ± 18.8	320.4 ± 18.4	318.4 ± 15.6	0.0555
Temporal Inner	314.2 ± 18.8	306.3 ± 15.8	307.5 ± 17.7	0.0638
Superior Inner	324.7 ± 19.4	319.7 ± 18.1	319.2 ± 17.0	0.7273
Inferior Inner	322.3 ± 18.5	316.7 ± 18.0	316.9 ± 16.5	0.2013
Nasal Outer	293.4 ± 19.0	292.1 ± 16.5	290.7 ± 13.2	0.7085
Temporal Outer	262.3 ± 16.7	258.4 ± 13.2	260.9 ± 17.3	0.5163
Superior Outer	277.0 ± 17.5	274.5 ± 14.2	275.2 ± 14.3	0.7273
Inferior Outer	264.1 ± 15.1	262.5 ± 14.0	267.5 ± 18.8	0.3284

For each of the nine areas specified in Table 2, a conventional one-way ANOVA was performed, in order to verify whether or not the thickness data alone can differentiate between the three groups under study. No significant main effects were found between the three groups, for any of the regions.

Regarding the classification, validation was carried out resorting to the *k*-fold cross-validation. *k* values of two, five and ten were used, where *k* = 2 represents the most conservative approach, and *k* = 5 and *k* = 10, the figures traditionally used, for comparison purposes only.

When the RNFL is ignored, the present analysis yields the best results. Therefore, presented results are based on the five of the six innermost layers, RNFL excluded—namely the GCL, IPL, INL, OPL and ONL layers.

Because the data partition (data split into training and testing sets) is random, and therefore distinct at each run, results are presented as distributions of performance indicators from 100 consecutive runs (Table 3).

**Table 3:** Distribution for the sensitivity (SEN) (%) and specificity (SPE) (%) for the healthy control (HC), Alzheimer’s disease (AD) and Parkinson’s disease (PD) groups, accuracy (ACC) (%), percentage of people with both eyes with the same classification (two eyes), percentage of correct classifications from the pool of people that received the same classification on both eyes (two eyes correct), and the percentage of eyes with a tie on the classification (unknown), for three *k* values (*k*-fold cross-validation).

		== HC ==		== AD ==		== PD ==		Accuracy	Two eyes	Two eyes correct	Unknown
		SEN	SPE	SEN	SPE	SEN	SPE				
*k* = 2	Max	90.6	89.2	82.1	94.4	87.0	100.0	82.9	81.7	96.2	4.1
	3^*rd*^ Q	86.8	81.7	74.4	92.5	77.8	97.8	78.8	73.2	92.7	2.1
	Median	84.9	80.6	71.8	90.7	74.1	96.7	76.7	69.0	91.4	1.4
	1^*st*^ Q	81.1	78.5	66.7	89.7	70.4	95.1	74.7	66.2	89.3	0.7
	Min	67.9	72.0	48.7	84.1	61.1	92.4	69.2	60.6	82.0	0.0
*k* = 5	Max	94.3	88.2	84.6	97.2	87.0	100.0	87.7	83.1	96.6	4.1
	3^*rd*^ Q	88.7	84.9	82.0	93.5	78.7	98.9	82.2	76.1	94.3	2.1
	Median	87.7	83.9	79.5	92.5	75.9	97.8	80.8	74.6	92.8	1.4
	1^*st*^ Q	84.9	81.7	74.4	90.6	74.1	96.7	79.5	73.2	91.9	0.7
	Min	77.4	76.3	69.2	86.9	66.7	94.6	75.3	64.8	87.0	0.0
*k* = 10	Max	96.2	88.2	84.6	96.3	85.2	100.0	86.3	81.7	96.6	3.4
	3^*rd*^ Q	90.6	86.0	82.1	93.5	79.6	98.9	83.6	77.5	95.9	2.1
	Median	88.7	84.9	79.5	92.5	77.8	97.8	82.2	76.1	94.4	1.4
	1^*st*^ Q	86.7	82.8	78.2	91.6	75.9	97.8	80.8	73.2	92.8	1.0
	Min	83.0	79.6	74.4	88.8	66.7	95.7	78.1	67.6	88.9	0.0

Only four of the features used in the classification process showed a strong correlation with the thickness of the respective layer and quadrant, with the correlation coefficient ranging from 0.70 to 0.73. Another 17 features showed a moderate correlation (greater than or equal to 0.50). None of the features presents a strong correlation in more than one of the three groups, and only two present a strong correlation in one group and a moderate correlation in another group. As a result, it is possible to state that texture features under analysis convey information on differences present in the retina that are not conveyed by thickness. For further information on the correlation values between these 21 features and thickness, refer to S3 Table.

The results obtained, even when considering the most conservative scenario (*k* = 2), clearly indicate that these biomarkers are useful in classifying cases into one of the three considered groups. Moreover, such biomarkers allow distinguishing between AD and PD eyes and patients. Additionally, these results state that, in just 1 to 2% of the cases, no differences seem to exist between the health status and either of the two neurodegenerative disorders considered in this work, nor between the two diseases themselves. Of particular importance is the case when the two eyes of the same subject received the same classification. In these conditions, 82% to 96% of the cases are correctly classified solely based on the analysis of the texture of the retina, as imaged by OCT.

Leaving out the RNFL, a total of 15 classification models were employed (three classification tests for each of the five remaining inner layers). While global texture metrics were used in 10 of these 15 models (66.7%), they represent only 16.7% of the total used features.

The analysis of the regional origin of the features that led to the above classification results shows an uneven contribution of the macular quadrants of the human retina. For the discrimination between the healthy control group and the AD group, over 65% (17 out of 26) features come from the superior macular region, seven from the superior-temporal quadrant and ten from the superior-nasal quadrant. Six and three features come from, respectively, the inferior-temporal and inferior-nasal quadrants. Additional four features come from the global texture.

Concerning the total number of features discriminating between the healthy control group and the PD group, there is an equal contribution from the superior and inferior regions of the retina. Similarly, there is an equal contribution from the temporal and nasal regions. Nevertheless, the superior-nasal and inferior-temporal macular regions contribute with only three features each (3/23), while the superior-temporal and inferior-nasal macular regions contribute, respectively, with ten and seven features. Moreover, seven features come from the global texture, almost twice that of the AD group.

## Discussion

Our results indicate that SVM, a supervised machine learning method, may aid in the concomitant clinical diagnosis of AD and PD, even in the absence of univariate differences on average thickness. The technique herewith presented demonstrates to be able not only in discriminating between eyes of HC and patients but also in distinguishing between the eyes of AD and PD patients. Furthermore, this method is based on the non-invasive imaging of the ocular fundus by OCT, a widely available technique, nowadays standard in private clinical practice. Particularly encouraging in terms of the reliability and reproducibility of the approach is the fact that the vast majority of people receiving the same classification on both eyes do, in fact, have the correct classification, with median percentages of 91.4 (2-fold cross-validation) up to 94.4 (10-fold cross-validation).

In this work, we used non-linear (radial basis function kernel) SVMs. They allow for the identification of relevant features that distinguish between any two of the three groups, and for the use of these features to classify cases into one of the three possible groups.

The six wavelet-based parameters used were computed from the six directionally-selective detail subband images, which were isolated from the lower-frequency (i.e. less detailed) image information at the first level of the wavelet decomposition process. Note that each of these subbands captures the original image’s details oriented at one out of six spatial orientations (±15°, ±45°, ±75°). As these parameters correspond to the variance of the magnitude of the complex wavelet coefficients of the respective subband, they represent a measure of the spread of the grey-level distribution of that same subband, and ultimately reflect the contrast of the image’s texture.

Global texture metrics were used in 66.7% of the classification models suggesting that these differences are spread over the entire macular region. Nevertheless, they represent only 16.7% of the total used features, which reinforces the need for local analysis to detect actual differences between health status and neurodegenerative diseases.

In the present work, the methodological research made on OCT data is far from its classical use, which may explain its discrimination power. While most of the reported works in the field rely on thickness measurements, texture data (from images computed per each retinal layer) were the metrics under evaluation here. In other words, our approach captures multivariate information, which is just not possible with simple thickness-based univariate comparisons. The inherent limitation of thickness-based approaches is likely a contributing factor to the inconsistent findings reported for both AD and PD. While several studies do not find thickness differences between HC and AD or PD patients [17, 19, 22], others report significant thinning of different retinal layers [9, 18, 20, 21]. To add to this inconclusiveness, when thickness differences between each of these two neurodegenerative disorders and the healthy condition are indeed reported, they typically concern retinal thickness measurements performed on patients of either disorder’s course at stages other than the initial stage.

Texture conveys information on the regular or irregular distribution of image intensity and, as such, it carries information on the structural arrangement of the different retinal layers and how they differ between the health, AD and PD conditions. Currently, the clinical diagnosis of these two neurodegenerative disorders is a challenging task, since there are no definite *in vivo* biomarkers. Texture analysis of OCT data may thus represent a novel tool in the identification of these diseases, providing a simple, inexpensive and non-invasive method of directly assessing neurodegeneration.

## Supporting information

S1 Fig Relation between patient age and disease duration for the Parkinson’s disease group. (TIF)

S2 Fig Diagram of the classification process. (TIF)

S1 Table MoCA (Montreal cognitive assessment) score results for the AD patients in the study group. (PDF)

S2 Table MoCA (Montreal cognitive assessment), UPDRS (unified Parkinson’s disease rating scale)—Motor and H&Y (Hoehn and Yahr) score results for the PD patients in the study group. (PDF)

S3 Table Features showing a moderate (0.5–0.7) and strong (> 0.7; highlighted in yellow) Pearson Correlation Coefficient (PCC) with thickness, per layer, per Q*i*—quadrant *i* (see Fig 2) for local features, and for each of the subject classes. In the features IMC1 and IMC2, IMC stands for Informal Measure of Correlation.
(PDF)

S1 File Full retina thickness, per sector, for all subjects. (XLSX)

S2 File Texture metrics for all subjects, quadrants, and layers. (XLSX)

## References

[1] Ferri CP, Prince M, Brayne C, Brodaty H, Fratiglioni L, Ganguli M, et al Global prevalence of dementia: a Delphi consensus study. Lancet. 2005; 366(9503):2112–2117. 10.1016/S0140-6736(05)67889-0 16360788 PMC2850264

[2] Alzheimer’s Association. 2016 Alzheimer’s disease facts and figures. Alzheimer’s and Dementia. 2016; 12(4):459–509. 27570871 10.1016/j.jalz.2016.03.001

[3] World Health Organization. Neurological disorders—public health challenges. 2006.

[4] Shah H, Albanese E, Duggan C, Rudan I, Langa KM, Carrillo MC, et al Research priorities to reduce the global burden of dementia by 2025. The Lancet Neurology. 2016; 15(12):1285–1294. 10.1016/S1474-4422(16)30235-6 27751558

[5] Masland RH. The neuronal organization of the retina. Neuron. 2012; 76(2):266–280. 10.1016/j.neuron.2012.10.002 23083731 PMC3714606

[6] London A, Benhar I, Schwartz M. The retina as a window to the brain—from eye research to CNS disorders. Nature Reviews Neurology. 2012 10.1038/nrneurol.2012.227 23165340

[7] Svetozarskiy SNN, Kopishinskaya SVV. Retinal Optical Coherence Tomography in Neurodegenerative Diseases (Review). Sovremennye tehnologii v medicine. 2015; 7(1):116–123. 10.17691/stm2015.7.1.14

[8] Cheung CYl, Ikram MK, Chen C, Wong TY. Imaging retina to study dementia and stroke. Progress in Retinal and Eye Research. 2017; 57:89–107. 10.1016/j.preteyeres.2017.01.001 28057562

[9] den Haan J, Verbraak FD, Visser PJ, Bouwman FH. Retinal thickness in Alzheimer’s disease: a systematic review and meta-analysis. Alzheimer’s and Dementia: Diagnosis, Assessment and Disease Monitoring. 2017; 6:162–170. 10.1016/j.dadm.2016.12.014 28275698 PMC5328759

[10] Hart NJ, Koronyo Y, Black KL, Koronyo-Hamaoui M. Ocular indicators of Alzheimer’s: exploring disease in the retina. Acta Neuropathologica. 2016; 132:767–787. 10.1007/s00401-016-1613-6 27645291 PMC5106496

[11] Archibald NK, Clarke MP, Mosimann UP, Burn DJ. The retina in Parkinson’s disease. Brain. 2009; 132:1128–1145. 10.1093/brain/awp068 19336464

[12] Tian T, Zhu XH, Liu YH. Potential role of retina as a progression of Parkinson’s disease. International Journal of Ophthalmology. 2011; 4(4):433–438. 10.3980/j.issn.2222-3959.2011.04.21 22553695 PMC3340867

[13] Ascaso F, Cruz N, Modrego P, Lopez-Anton R, Santabárbara J, Pascual L, et al Retinal alterations in mild cognitive impairment and Alzheimer’s disease: an optical coherence tomography study. Journal of Neurology. 2014; 261:1522–1530. 10.1007/s00415-014-7374-z 24846203

[14] Cheung CYL, Ong YT, Ikram MK, Ong SY, Li X, Hilal S, et al Microvascular network alterations in the retina of patients with Alzheimer’s disease. Alzheimer’s and Dementia. 2014; 10(2):135–142. 10.1016/j.jalz.2013.06.009 24439169

[15] Chorostecki J, Seraji-Bozorgzad N, Shah A, Bao F, Bao G, George E, et al Characterization of retinal architecture in Parkinson’s disease. Journal of the Neurological Sciences. 2015; 355:44–48. 10.1016/j.jns.2015.05.007 26071887

[16] Garcia-Martin E, Larrosa JM, Polo V, Satue M, Marques ML, Alarcia R, et al Distribution of retinal layer atrophy in patients with Parkinson disease and association with disease severity and duration. American Journal of Ophthalmology. 2014; 157(2):470–478. 10.1016/j.ajo.2013.09.028 24315296

[17] Aaker GD, Myung JS, Ehrlich JR, Mohammed M, Henchcliffe C, Kiss S. Detection of retinal changes in Parkinson’s disease with spectral-domain optical coherence tomography. Clinical Ophthalmology. 2010; 4:1427–1432. 10.2147/OPTH.S15136 21188154 PMC3000768

[18] Altintaş Ö, Işeri P, Özkan B, Çağlar Y. Correlation between retinal morphological and functional findings and clinical severity in Parkinson’s disease. Documenta Ophthalmologica. 2008; 116:137–146. 10.1007/s10633-007-9091-8 17962989

[19] Archibald NK, Clarke MP, Mosimann UP, Burn DJ. Retinal thickness in Parkinson’s disease. Parkinsonism and Related Disorders. 2011; 17:431–436. 10.1016/j.parkreldis.2011.03.004 21454118

[20] Hajee ME, March WF, Lazzaro DR, Wolintz AH, Shrier EM, Glazman S, Bodis-Wollner IG. Inner retinal layer thinning in Parkinson disease. Archives of Ophthalmology. 2009; 127(6):737–741. 10.1001/archophthalmol.2009.106 19506190

[21] Garcia-Martin E, Bambo MP, Marques ML, Satue M, Otin S, Larrosa JM, et al Ganglion cell layer measurements correlate with disease severity in patients with Alzheimer’s disease. Acta Ophthalmologica. 2016; 94:e454–e459. 10.1111/aos.12977 26895692

[22] Lad EM, Mukherjee D, Stinnett SS, Cousins SW, Potter GG, Burke JR, et al Evaluation of inner retinal layers as biomarkers in mild cognitive impairment to moderate Alzheimer’s disease. PLoS ONE. 2018; 13(2):e0192646 10.1371/journal.pone.0192646 29420642 PMC5805310

[23] Bernardes R, Silva G, Chiquita S, Serranho P, Ambrósio AF. Retinal biomarkers of Alzheimer’s disease: insights from transgenic mouse models. In: 14th International Conference on Image Analysis and Recognition (ICIAR); 2017.

[24] Nunes A, Ambrósio AF, Castelo-Branco M, Bernardes R. Texture biomarkers of Alzheimer’s disease and disease progression in the mouse retina. 18th International Conference on Bioinformatics and Bioengineering (BIBE). 2018;.

[25] Anantrasirichai N, Achim A, Morgan JE, Erchova I, Nicholson L. SVM-based texture classification in optical coherence tomography. IEEE 10th International Symposium on Biomedical Imaging: From Nano to Macro. 2013.

[26] Mohammad S. Textural measurements for retinal image analysis. University of Manchester. 2014. Available from: https://www.research.manchester.ac.uk/portal/files/54570132/FULL_TEXT.PDF.

[27] Gao W. Improving the quantitative assessment of intraretinal features by determining both structural and optical properties of the retinal tissue with optical coherence tomography. University of Miami. 2012. Available from: https://scholarlyrepository.miami.edu/oa_dissertations/855.

[28] González A, Remeseiro B, Ortega M, Penedo MG, Charlón P. Automatic cyst detection in OCT retinal images combining region flooding and texture analysis. IEEE International Symposium on Computer-Based Medical Systems. 2013.

[29] Kassner A, Thornhill RE. Texture analysis: A review of neurologic MR imaging applications. American Journal of Neuroradiology. 2010; 31(5):809–816. 10.3174/ajnr.A2061 20395383 PMC7964174

[30] Haralick RM, Shanmugam K, Dinstein I. Texture features for image classification. IEEE Transactions on Systems, Man and Cybernetics. 1973; SMC-3(6):610–621. 10.1109/TSMC.1973.4309314

[31] Tomita F, Tsuji S. Computer analysis of visual textures. Springer 1990.

[32] World Medical Association. Declaration of Helsinki—ethical principles for medical research involving human subjects. Journal of the American Medical Association. 2013; 310(20):2191–2194. 10.1001/jama.2013.281053 24141714

[33] McKhann GM, Knopman DS, Chertkow H, Bradley HT, Clifford JJR, Kawas CH, et al The diagnosis of dementia due to Alzheimer’s disease: Recommendations from the National Institute on Aging—Alzheimer’s Association workgroups on diagnostic guidelines for Alzheimer’s disease. Alzheimer’s & Dementia. 2011; 7(3):263–269. 10.1016/j.jalz.2011.03.005 PMC3312024 21514250

[34] Nasreddine ZS, Phillips NA, Béridian V, Charbonneau S, Whitehead V, Collin I, et al The Montreal cognitive assessment, MoCA: a brief screening tool for mild cognitive impairment. Journal of the American Geriatrics Society. 2005; 53:695–699. 10.1111/j.1532-5415.2005.53221.x 15817019

[35] Berg L. Clinical Dementia Rating (CDR). Psychopharmacology Bulletin. 1988; 24(4):367–369. 3249765

[36] Leuzy A, Chiotis K, Hasselbalch SG, Rinne JO, De Mendonça A, Otto M, et al Pittsburgh compound B imaging and cerebrospinal fluid amyloid-beta in a multicentre European memory clinic study. Brain. 2016; 139(9):2540–2553. 10.1093/brain/aww160 27401520 PMC4995359

[37] Hughes AJ, Daniel SE, Kilford L, Lees AJ. Accuracy of clinical diagnosis of idiopathic Parkinson’s disease: A clinico-pathological study of 100 cases. Journal of Neurology Neurosurgery and Psychiatry. 1992; 55:181–184. 10.1136/jnnp.55.3.181 PMC1014720 1564476

[38] Guimarães P, Rodrigues P, Lobo C, Leal S, Figueira J, Serranho P, et al Ocular fundus reference images from optical coherence tomography. Computerized Medical Imaging and Graphics. 2014; 38:381–389. 10.1016/j.compmedimag.2014.02.003 24631317

[39] Yazdi M, Gheysari K. A new approach for the fingerprint classification based on gray-level co-occurrence matrix. World Academy of Science, Engineering and Technology. 2008; 47.

[40] Clausi DA. An analysis of co-occurrence texture statistics as a function of grey level quantization. Canadian Journal of Remote Sensing. 2002; 28(1):45–62. 10.5589/m02-004

[41] Maheshwari S, Pachori RB, Acharya UR. Automated diagnosis of glaucoma using empirical wavelet transform and correntropy features extracted from fundus images. IEEE Journal of Biomedical and Health Informatics. 2017; 21(3). 10.1109/JBHI.2016.2544961 28113877

[42] Häfner M, Kwitt R, Uhl A, Gangl A, Wrba F, Vécsei A. Feature extraction from multi-directional multi-resolution image transformations for the classification of zoom-endoscopy images. Pattern Analysis and Applications. 2009; 12:407–413. 10.1007/s10044-008-0136-8

[43] Wimmer G, Tamaki T, Tischendorf JJW, Häfner M, Yoshida S, Tanaka S, et al Directional wavelet based features for colonic polyp classification. Medical Image Analysis. 2016; 31:16–36. 10.1016/j.media.2016.02.001 26948110

[44] Etehadtavakol M, Ng EYK, Chandran V, Rabbani H. Separable and non-separable discrete wavelet transform based texture features and image classification of breast thermograms. Infrared Physics & Technology. 2013; 61:274–286. 10.1016/j.infrared.2013.08.009

[45] Jian W, Sun X, Luo S. Computer-aided diagnosis of breast microcalcifications based on dual-tree complex wavelet transform. BioMedical Engineering Online. 2012; 11(96). 10.1186/1475-925X-11-96 23253202 PMC3537591

[46] Selesnick IWW, Baraniuk RGG, Kingsbury NCC. The dual-tree complex wavelet transform. IEEE Signal Processing Magazine. 2005; p. 123–151. 10.1109/MSP.2005.1550194

[47] Celik T, Tjahjadi T. Multiscale texture classification using dual-tree complex wavelet transform. Pattern Recognition Letters. 2009; 30:331–339. 10.1016/j.patrec.2008.10.006

[48] Wang S, Lu S, Dong Z, Yang J, Yang M, Zhang Y. Dual-tree complex wavelet transform and twin support vector machine for pathological brain detection. Applied Sciences. 2016; 6(169).

[49] Duda RO, Hart PE, Stork DG. Pattern classification. 2nd ed Wiley-Interscience 2000.

[50] Chang CC, Lin CJ. LIBSVM: a library for support vector machines ACM Transactions on Intelligent Systems and Technology. 2011; 2(3(27)).

