# Supplementary figures and images for: Retinal texture biomarkers may help to discriminate between Alzheimer’s, Parkinson’s, and healthy controls
Source: PLoS One. 2019 Jun 21;14(6):e0218826. doi: 10.1371/journal.pone.0218826 (PMC6588252; doi:10.1371/journal.pone.0218826)

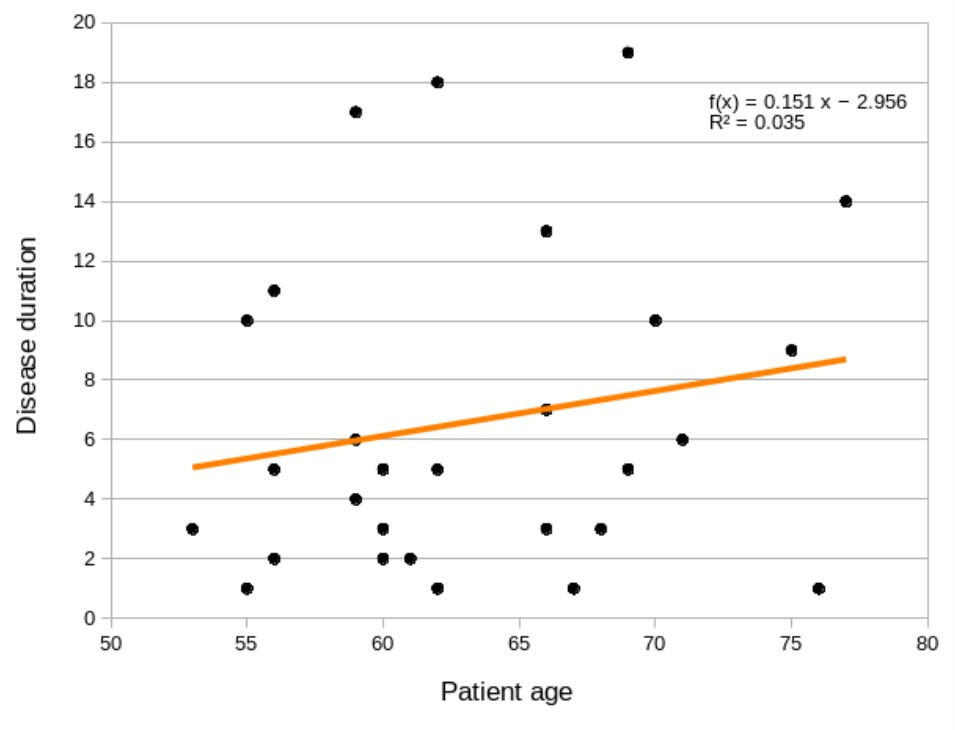

Supplement: S1 Fig — (TIF) [file pone.0218826.s001.tif]

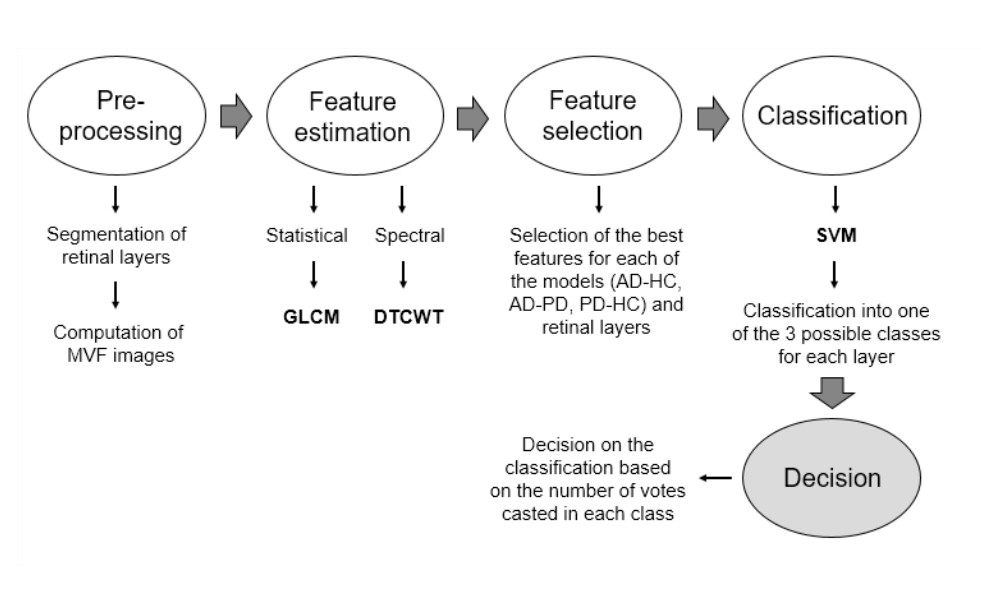

Supplement: S2 Fig — (TIF) [file pone.0218826.s002.tif]
