# Supplementary material for: Retinal texture biomarkers may help to discriminate between Alzheimer’s, Parkinson’s, and healthy controls
Source: PLoS One. 2019 Jun 21;14(6):e0218826. doi: 10.1371/journal.pone.0218826 (PMC6588252; doi:10.1371/journal.pone.0218826)
Supplement: S1 Table — (PDF) [file pone.0218826.s003.pdf]

#	**Eyes**	**Age**	**Gender**	**Disease duration**	**MoCA**
1	OD/OS	76	F	1	7
2	OD/OS	76	M	1	21
3	OD/OS	73	F	1	7
4	OD/OS	72	M	1	14
5	OD/OS	72	M	1	13
6	OD/OS	72	F	0	12
7	OD/OS	71	F	0	9
8	OD/OS	70	F	2	16
9	OD/OS	69	F	0	16
10	OD/OS	68	F	0	10
11	OD/OS	68	M	1	19
12	OD/OS	66	M	0	18
13	OD/OS	65	M	1	14
14	OD/OS	63	F	1	17
15	OD/OS	61	M	1	16
16	OD/OS	60	F	1	10
17	OD/OS	58	M	0	17
18	OD/OS	56	F	0	17
19	OD/OS	55	M	0	21
20	OD	54	M	0	13
