# Supplementary material for: Retinal texture biomarkers may help to discriminate between Alzheimer’s, Parkinson’s, and healthy controls
Source: PLoS One. 2019 Jun 21;14(6):e0218826. doi: 10.1371/journal.pone.0218826 (PMC6588252; doi:10.1371/journal.pone.0218826)
Supplement: S2 Table — (PDF) [file pone.0218826.s004.pdf]

#	**Eyes**	**Age**	**Gender**	**Disease duration**	**MoCA**	**UPDRS – Motor**	**H&Y**
1	OD/OS	69	M	5	24	28	2
2	OD/OS	67	F	1	9	22	1
3	OD/OS	68	M	3	9	18	1.5
4	OD/OS	66	M	3	24	22	2
5	OD/OS	62	F	1	14	23	1
6	OD/OS	61	F	2	9	28	1
7	OD/OS	60	F	3	22	20	1
8	OD/OS	60	F	5	23	28	1
9	OD/OS	56	F	2	NA	25	NA
10	OD/OS	56	F	5	31	23	2.5
11	OD/OS	77	M	14	22	43	3
12	OD/OS	75	M	9	27	35	3
13	OD	71	F	6	10	44	2.5
14	OD/OS	70	F	10	11	44	3
15	OD/OS	69	F	19	21	19	2
16	OD/OS	66	M	13	27	35	2
17	OD/OS	62	M	5	24	27	2
18	OD/OS	59	M	6	27	2	0
19	OD/OS	55	M	10	23	16	1.5
20	OD/OS	53	F	3	21	29	2
21	OD/OS	66	F	7	25	28	1
22	OD/OS	59	M	4	45	22	2
23	OD/OS	56	M	11	32	20	2
24	OD/OS	55	M	1	14	22	1
25	OD/OS	76	F	1	12	33	2
26	OD/OS	62	F	18	23	19	2
27	OS	60	M	2	22	12	1
28	OD/OS	59	F	17	25	21	2
