# Supplementary material for: Retinal texture biomarkers may help to discriminate between Alzheimer’s, Parkinson’s, and healthy controls
Source: PLoS One. 2019 Jun 21;14(6):e0218826. doi: 10.1371/journal.pone.0218826 (PMC6588252; doi:10.1371/journal.pone.0218826)
Supplement: S3 Table — In the features IMC1 and IMC2, IMC stands for Informal Measure of Correlation. (PDF) [file pone.0218826.s005.pdf]

	Alzheimer's disease		Parkinson's disease		Healthy controls	
GCL	Difference Variance (Q2)	0.56	Correlation (Q1)	0.53	IMC2 (Q4)	0.52
	Dissimilariy (Q2)	0.57	Sum Entropy (Q1)	0.57	Variance +15° (Global)	0.64
	Inertia (Q2)	0.56			Variance -75° (Global)	0.68
IPL	Correlation (Q1)	0.58	Cluster Shade (Q1)	0.64	Cluster Shade (Q1)	0.51
	Variance +75° (Global)	0.73	Cluster Shade (Q2)	0.72	Correlation (Q1)	0.71
	Variance -75° (Global)	0.69	Uniformity (Q1)	0.72	Variance +15° (Global)	0.68
			Cluster Shade (Q4)	0.55	Variance -15° (Global)	0.67
			Entropy (Q4)	0.60	Variance +75° (Global)	0.64
			Sum Average (Q4)	0.59	Variance -75° (Global)	0.64
			Sum Entropy (Q4)	0.51		
INL	Variance +15° (Global)	0.54	Cluster Shade (Q1)	0.64		
	Variance -15° (Global)	0.56				
OPL	Sum Entropy (Q4)	0.51			Cluster Prominence (Q2)	0.50
	Variance +15° (Global)	0.57			IMC1 (Q2)	0.52
	Variance -15° (Global)	0.55			IMC2 (Q2)	0.58
ONL	IMC2 (Q1)	0.50				
